# Passive blood anaphylaxis: subcutaneous immunoglobulins are a cause of ongoing passive anaphylactic reaction

**DOI:** 10.1186/s13223-017-0213-x

**Published:** 2017-09-15

**Authors:** Przemyslaw Zdziarski, Andrzej Gamian, Jacek Majda, Agnieszka Korzeniowska-Kowal

**Affiliations:** 1 Department of Clinical Immunology, Lower Silesian Center for Cellular Transplantation, POBox 1818, 50-385 Wrocław-46, Poland; 2 Department of Immunology of Infectious Diseases, Hirszfeld Institute of Immunology and Experimental Therapy, Polish Academy of Sciences, POBox 1818, 50-385 Wrocław-46, Poland; 3 Department Laboratory, 4th Military Teaching Hospital, Wrocław, Poland

**Keywords:** Subcutaneous immunoglobulins (ScIg), IgE, IgG, Serum half-life, Anaphylaxis, Hypersensitivity, Prausnitz–Küstner reaction, Adverse drug reactions (ADRs), Transfusion, Case report

## Abstract

**Background:**

Allergic, especially anaphylactic, reactions during immunoglobulin replacement therapy are rare, but their pathophysiology and classification remain ambiguous. Recent findings show positive results of skin tests with commercially available immunoglobulins, but target antigens and responsible compounds of the tested immunoglobulins have not been strictly identified.

**Case description and findings:**

Four adult patients with recently diagnosed common variable immunodeficiency qualified for standard subcutaneous immunoglobulin replacement therapy regimen. They had no history of receiving immunoglobulins, blood or blood product transfusions. Edema, confluent wheals and erythema were observed at the site of subcutaneous immunoglobulin infusion: typical early and late phase reaction. A transient increase in various passively transferred IgG and IgE antibodies was responsible for misleading positive outcome of the serological testing for active humoral response such as type I allergy, anti-Rh, isohemagglutinins and rheumatoid factor (RF). Although the clinical presentation was very unusual and severe, the retrospective analysis showed no isohemagglutinins, RF and IgE in the patients’ serum before but it was positive after the infusion (median IgE = 18 IU/ml, RF = 8 IU/ml). Type I allergic reaction (laryngeal edema, rhinoconjuctivitis) came out at +14 days of replacement therapy when the patient visited countryside. In the second patient anaphylactic reaction was observed 5 days after ScIg administration, and only when the patient consumed peanuts. Therefore, IgE concentration was measured retrospectively in a series of commercial preparations used in the initial subcutaneous immunoglobulin replacement therapy that caused the adverse event (AE) and it was determined between 138 and 232 IU/ml (kU/l), i.e. 690–2100 IU per g of protein. Specific IgE was within a wide range from 198 (mix of food) to 2809 kUA/l (mix of grass) but many of the tested allergen-specific IgE were class 2 or 3 (i.e. 0.71–17.5 kUA/l).

**Conclusions:**

The case resembles passive cutaneous anaphylaxis and Prausnitz–Küstner reaction but clinical significance of the classical phenomena has not yet been described. This observation indicates that anaphylactic reactions during immunoglobulin replacement therapy may result from IgE or pathological IgG content. Such IgE presence was sporadically reported (34.5–105 IU/ml, i.e. 862.5–1450 IU/g of protein) in intravenous immunoglobulins that are used and monitored by healthcare professionals. In clinical practice the definition of adverse events is inadequate since individual batches of immunoglobulins come with different specificity therefore, they should be classified as transfusion products (not bioequivalents). Such new approach implies establishing (1) new control methods and strategies to ensure introduction of the safety regulations for subcutaneous home self-administration of immunoglobulins as well as (2) guidelines for the prevention of anaphylaxis in patients receiving immunoglobulins (for example peanut).

## Background

The choice of methods of drug production, their further purification, sterilization, distribution and storage are critical to pharmacovigilance. Immunoglobulin preparations are of a paramount importance to management of the primary humoral immunodeficiency. Subcutaneous immunoglobulin (ScIg) infusion as replacement therapy is frequently chosen for patients with primary antibody deficiencies (PAD). Although a routine immunoglobulin therapy involves intramuscular injections, it has nowadays been replaced by subcutaneous infusion. Commercially available immunoglobulin preparations contain IgG antibodies, pooled from >3000 donors, with heterogeneous specificity and physicochemical properties [1] as well as trace amounts of IgA, sometimes human albumin, but according to product specifications they should be free of IgM and IgE. Therefore, purification, standardization and quality control of the therapeutic antibodies are of a great importance.

Despite progress in identification in the immunoglobulins preparations of the basic components believed to cause hypersensitivity, the basic mechanisms of these phenomena remain elusive. Not surprisingly, only a broad contraindication specified in manufacturer’s leaflets as well as product characteristics concerns cases of *hypersensitivity to any compound* (according to common WHO/NIH terminology of adverse drug reactions). Most of the immunoglobulin product descriptions do not specify frequency of anaphylaxis. At the same time, anti-IgA immune response is over-diagnosed as the cause of anaphylaxis [2, 3]. It is of great significance that most serious adverse events are observed during the first immunoglobulin administration in patients with primary antibody deficiency: untreated patients receiving their first infusion are at most risk of adverse reaction [4] since their specific antibodies must invariably be very low with absent immunization responses as well as absent isohemagglutinins (ESID criteria for CVID).

The aim of this report is to shed light on new mechanisms of hypersensitivity/anaphylactic reaction occurring during initial immunoglobulin replacement therapy.

## Case description

**Fig. 1:**
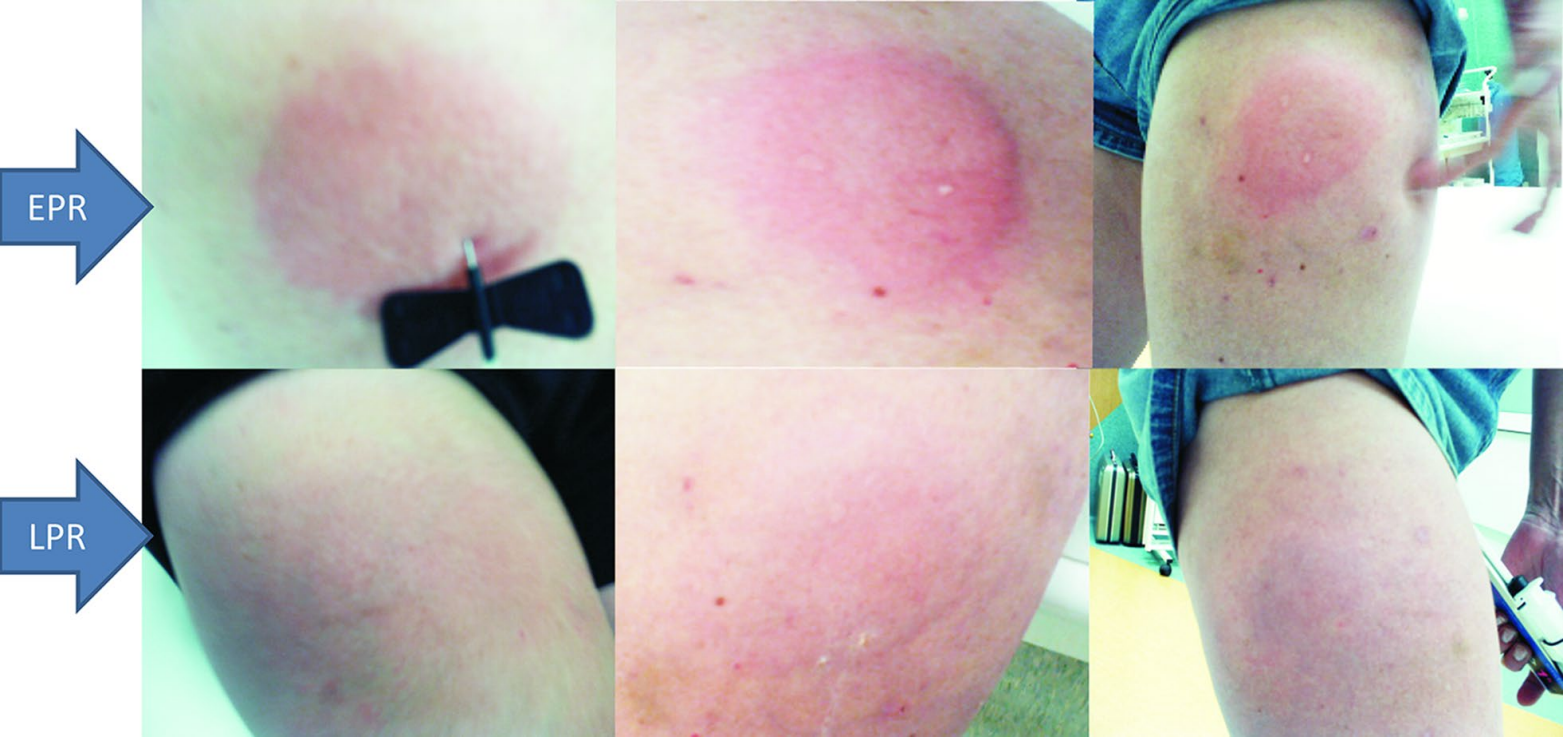
Early (EPR, *top panel*) and late (LPR, *bottom panel*) phase reaction respectively 0, 5 and 6 h after injection of IgE-contained ScIg

**Fig. 2:**
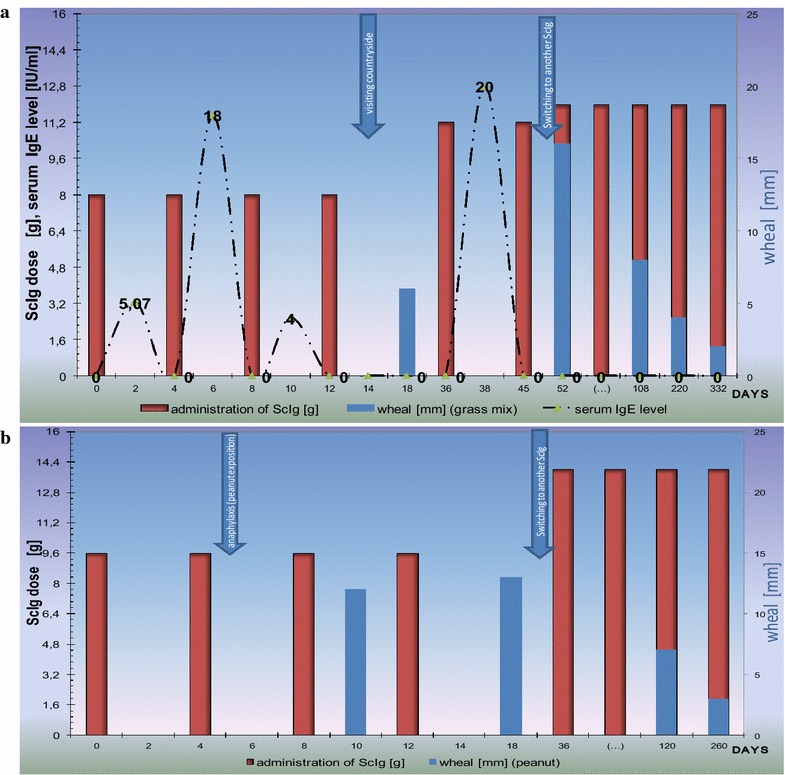
Passive type I (anaphylactic) reaction after ongoing sensitization during initial subcutaneous immunoglobulins repacement therapy. Timeline of two CVID patients receiving initial ScIg therapy. Negative skin test before, but positive after replacement therapy with Subcuvia^®^ was presented. **a** Patient with rhinoconjunctivitis and laryngeal edema occurring after exposure to grass pollens. Serum IgE level fluctuation was presented. It was tested retrospectively every 2 days when ScIg loading dose was given over the course of 2 weeks. Serum IgE level and short serum half-life do not reflect IgE elimination, but FcƐR expression and opsonization of immune cells that are source positive skin tests. **b** Patient with anaphylaxis after peanut exposition during home-based self-administration of ScIg. ScIg home administration, and “take peanut home” messages of guidelines [28] may be the cause of anaphylactic complication. Noteworthy, still positive test with the gradual decrease of wheal was observed after switching ScIg product (Subcuvia^®^ withdrawal) to another one (Hizentra^®^). The first product contains IgE, the other—does not

Adult patients (aged 30–50 years) with diagnosed common variable immunodeficiency (CVID) according to ESID criteria and clinical characteristics [5] were referred for standard immunoglobulin replacement therapy. Due to professional activity (frequent traveling) or poor venous access, four of them were qualified for subcutaneous (ScIg) infusion (following recommendations in the product specification of Subcuvia^®^). They had no history of receiving immunoglobulins, blood or blood product transfusions. Neither hypersensitivity reactions nor allergy (especially to latex, amoxicillin, benzyl penicillin, grass pollens and peanuts) were observed in the natural history of the disease. Antibody of the IgE class was not detected even in patients with the giardiasis, that normally prompts a very high level of IgE in immunocompetent subjects [6]. If IgE is shown to be absent in such cases, active IgE-mediated immune response and anaphylactic reactions can essentially be excluded. Three of the patients, before being diagnosed in our center with CVID, showed negative results for common allergens, when using skin prick test. Delayed hypersensitivity test with PPD was negative for all four patients. Patients received ScIg daily dose of 0.15 g/kg body weight (i.e. maximal daily dose of Subcuvia^®^), what corresponds to recommended dose of 400 mg/kg body weight per month according to the International Union of Immunological Societies Scientific Committee Guidelines, EMEA [7, 8] and to cumulative monthly dose in the order of 0.4–0.8 g/kg, as recommended in the product specification. Twelve minutes after the first ScIg injection (different batch of the product for each patient) edema, confluent wheal and erythema occurred (Fig. 1, top panel). The same reaction was observed in another area of the subcutaneous injection. A typical late phase reaction occurred 4–6 h later (Fig. 1, bottom panel, different batch of the product for each patient). One patient exhibited type I allergic reaction but 5 days after ScIg administration, rapid rhinoconjunctivitis and stridor (laryngeal edema) occurred after patient’s exposure to grass pollens (when patient visited countryside) (first representative patient after consent for SPT and further evaluation—Fig. 2a).

In spite of a good history of tolerance to peanuts, life-threatening anaphylactic symptoms (hypotension and then urticaria) appeared when the second patient ate peanuts (about 20 min after consumption and 5 days after the first ScIg infusion that happened to contain IgE, see Tables 2, 3, 4). Standard epinephrine administration plus first generation of parenteral antihistamine drug (clemastine) were successfully administered outside of our hospital (serum β-tryptase level was not tested). For secondary prevention and avoidance of inciting agents the skin prick (SPT) or intracutaneous tests (ICT) were performed. Initial negative results for IgE in patients’ sera became positive after ScIg infusions (Table 1). The ICT test was performed for the second representative patient too. SPT and ICT (4 and 8 months later, respectively) were still positive with peanut (Fig. 2b). At the injection site the ICT was higher than at other sites (wheal—12 mm vs 7 mm, flare—50 vs 30 mm, respectively). Both patients were successfully switched to another ScIg (Hizentra^®^) product without IgE content, wheal and LPR previously observed but substitution was made under medical supervision. Other two patients who withdrew the consent for further ScIg therapy were switched from home-based to hospital-based IVIg therapy. They were not enrolled for further evaluation.

**Table 1:** The IgG, IgM, IgA, IgE level in serum of four patients before and 4 days after initial ScIg administration of Subcuvia^®^

	IgG (mg/dl)	IgA (mg/dl)	IgM (mg/dl)	IgE (kU/l)	Specific antibody
Patients (before ScIg)					
Patient 1	58.0	<5.5	34.0	<0.35	RF (−) Isohaemagglutinins (−) Coombs tests (−)
Patient 2	182.0	<5.5	32.2	<0.35	
Patient 3	252.0	<5.5	5.0	<0.35	
Patient 4	269.0	<5.5	61.2	<0.35	
Median	217	<5.5	33.1	<5	
Patients (+4 days after ScIg) Coombs tests (+)					
Patient 1	426.0	<5.5	32.2	0.5	RF-12
Patient 2	585.0	22.0	27.8	14.0	RF-11
Patient 3	354.0	22.0	4.8	22.0	RF-5
Patient 4	396.0	25.0	59.8	16.0	RF-4
Median	411.0	22.0	30.0	18.0	RF-8 IU/ml

### Analysis of the patients’ serum samples

Blood samples were drawn before and during initial replacement therapy and serum immunoglobulin levels were determined retrospectively by turbidimetry (Olympus) [8]. Injection of immunoglobulin preparations containing pathological components (Tables 2, 3, 4), gave a transitory rise in the patient’s blood of various passively transferred antibodies, i.e. IgE, rheumatoid factor—RF, anti-Rh, anti-A, anti-B blood group isohemagglutinins (Table 1; Fig. 2a). Initial negative results for IgE in patients’ sera became temporarily positive after ScIg infusions (Table 1; Fig. 2a). Serum of first representative patient was collected during hospital-based initial replacement therapy and tested retrospectively. The timeline and IgE serum level during cumulative ScIg substitution are presented in Fig. 2a. Interestingly, fast decrease of serum IgE to below borderline level (i.e. <0.35 kU/l by the immunocap system) was observed at +4 days after each transfusion of Subcuvia^®^.

**Table 2:** Screening test of subcutaneous immunoglobulin used in the study

Batch	IgG (mg/dl)	IgA (mg/dl)	IgM (mg/dl)	IgE (IU/ml)	Other test and compounds (not described in specification)
VNG1F019	152,00	234	5.34	148	Albumin—22.4 mg/ml (14%)
VNG1G02	153,00	253	<17.7	232	Albumin—22.0 mg/ml (13.75%)
VNG1G011AB	150,00	308	<17.7	223	κ-3370 λ-1440
VNG1G004	151,00	259	4.39	138	RF-19.9

Drug specification of Subcuvia^®^: total protein—160 mg/ml; 95% immunoglobulins, i.e. 152 mg/ml = 15200 mg/dl; IgA < 4.8 mg/ml (480 mg/dl)

**Table 3:** Screening panels for specific IgE (UNICAP 100 PHARMACIA) in Subcuvia^®^ (Batch VNG1G02)

Screening panel	Specific IgE	Allergens	Result [kUA/l]
Food allergy—screening panels	fx10 (f26,f27,f75,f83, f284)	Pork, beef, egg yolk, chicken, Turkey	81
	fx5 (f1,f2,f3,f4,f13,f14)	Egg white, milk, fish, wheat, peanut, soya bean	198
Tree pollens—mix allergens	tx5 (t2, t4, t8, t12, t14)	*Alnus incana*, *Corylus avellana*, *Ulmus americana*, *Salix caprea*, *Populus deltoides*	914
	tx6 (t1, t3, t5, t7, t10)	*Acer negundo*, *Betula verrucosa*, *Fagus grandifolia*, *Quercus alba*, *Juglans californica*	852
Grass pollens—mix allergens	gx1 (g3, g4, g5, g6, g8)	*Dactylis glomerata*, *Festuca elatior*, *Lolium perenne*, *Phleum pratense*, *Poa pratensis*	2809
Weed pollens—mix allergens	wx3 (w6,w9,w10,w12,w20)	*Artemisia vulgaris*, *Plantago lanceolata*, *Chenopodium album*, *Solidago virgaurea*, *Urtica dioica*	256
Microorganisms—mix allergens	mx2 (m1,m2,m3,m5,m6,m8)	*Penicillium notatum*, *Cladosporium herbarum*, *Aspergillus fumigatus*, *Candida albicans*, *Alternaria alternata*, *Helminthosporium*	268

**Table 4:** Specific IgE to separate allergens in Subcuvia^®^ sample (Batch VNG1G02)

Allergen	IgE (kUA/l)	Class
*Dactylis glomerata*	1.78	2
*Festuca elatior*	2.05	2
*Lolium perenne*	0.52	1
*Phleum pratense*	1.46	2
*Poa pratensis*	0.40	1
*D. pteronyssimus*	3.42	2
*D. farine*	3.56	3
*Betula verrucosa*	1.16	2
*Corylus avellana*	0.69	1
*Populus deltoides*	1.78	2
*Artemisia vulgaris*	0.40	1
*Plantago lanceolata*	0.62	1
*Penicillium notatum*	0.80	2
*Cladosporium herbarum*	<0.35	0
*Candida albicans*	0.45	1

### ScIg product analysis

Samples of the Ig preparations, to be representative of what the patients received were collected before the end of the infusion and preserved for further investigations [9]. IgE analysis (screening panels and specific for each allergen) were performed with ImmunoCAP 100 PHARMACIA (Pharmacia & Upjohn Diagnostics AB, Uppsala, Sweden) in several drug samples (replacement immunoglobulin preparations). Total and specific IgE and other components were detected in several samples and presented in Tables 2, 3 and 4. Elevated values were observed for grass pollens (g × 1 mix allergens) and tree pollens (t × 5, t × 6 mix allergens) (Table 2). Type 1 reaction in patients corresponds to specific IgE observed in ScIg (Table 3), especially class 2 or 3 (listed in Tables 3, 4) in first patient.

## Discussion

To achieve the highest level of safety and quality of drugs the regulation experts recommend a stepwise approach. Unfortunately, immunoglobulins and other blood-derived products are not typical drugs, since plasma processing into various types of products is highly specialized. Large-scale processing (fractionation) is very important for ensuring quality and safety profiles of the products [10].

**Fig. 3:**
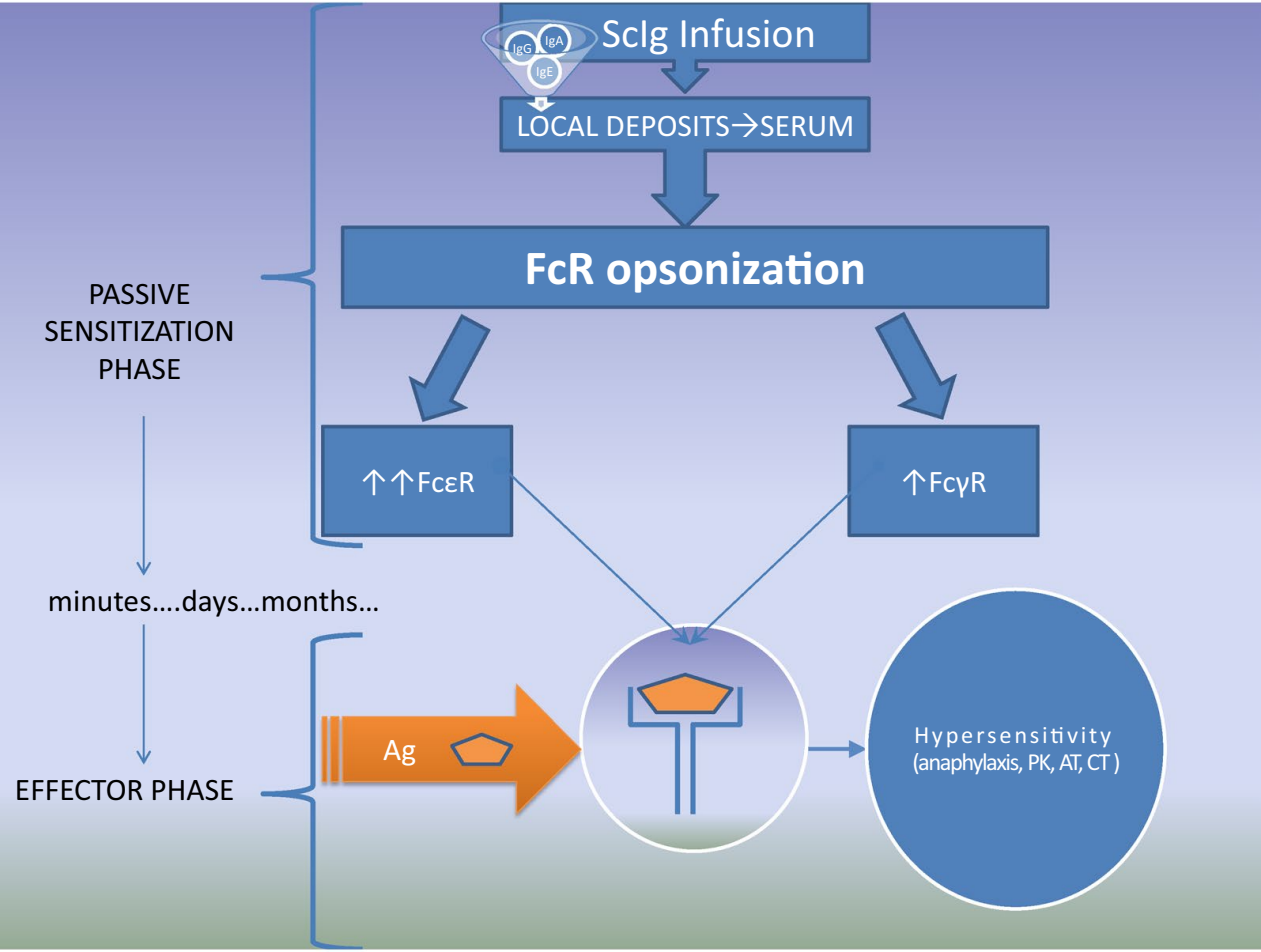
Role of passive transfer of pathologic IgE and IgG in ongoing passive anaphylactic reaction during subcutaneous immunoglobulin therapy. ScIg contamination observed in the study by IgE, anti-blood group and rheumatoid factor (RF) are the source of passive hypersensitivity. In the sensitization phase the initial ScIg administration (containing IgG, IgM and IgE see Tables 2, 3) forms a local depot and local opsonization of immune cells (see Fig. 1) to form Prausnitz–Küstner (PK) (IgE and FcεR), Arthus-type reaction (AR) (IgG and RF) or positive Coombs tests (CT). After absorption and transport through the lymphatic system, free immunoglobulins from serum opsonise the blood and peripheral compartment cells thereby increasing FcR expression [23, 24], i.e. 32-fold elevation in FcεRI expression [25]. The cell surface immunoglobulins may be the source of positive skin tests and hypersensitivity reaction after antigen/allergen stimulation (a few days/months later see Fig. 2). Noteworthy basophils are players in the IgG- but not IgE-mediated systemic anaphylaxis [20]

Many publications and ScIg product descriptions refer to “local tissue reaction” without more specific information about the reaction time (immediate, late), mechanism (type 1, 2 or 3; IgE or IgG-dependent) and frequency [11–14]. In contrast to typical drugs, immunoglobulins have different individual properties, the batches are not comparable. So, the pathomechanism of systemic anaphylactic reaction may depend on each specific batch (Table 2). It is noteworthy that blood products may elicit life-threatening graft-vs-host disease in patients with cellular immunodeficiency and anaphylaxis in those with humoral immunodeficiency, e.g. after hematopoietic stem cell transplantation (HSCT) [4]: observed here positive Coombs test (Table 1) has a tendency to increase the hypersensitivity reaction during immunoglobulin replacement therapy (Fig. 3).

The body’s reaction to administered blood-derived products depend on the duration of the patient’s exposure to the product (Fig. 2), type of administration (Figs. 1, 3) and nature of the antigen. When the exposure to allergen follows the infusion, inflammatory symptoms are observed at the time of contact (e.g. presented here type I and anaphylactic reaction). Noteworthy, recent guidelines for prevention of peanut allergy specific strategies for infants (as early as 4–6 months) at various levels of risk [15]: infants who develop wheal 3–7 mm in diameter should undergo oral challenge test at a specialist’s office. The history of the second patient indicates that the same recommendations should be introduced for children and patients with primary immunodeficiency after initial immunoglobulin replacement therapy even though they were previously SPT/ICT negative and showed good tolerance of peanut. The new unexpected adverse reaction is not consistent with applicable product information or characteristics of the drug.

The positive ICT for peanuts was observed at the injection site, as well as in other areas, but the level of the reaction differed significantly (wheal 12 vs 7 mm respectively). ScIg initially forms a local depot by non-covalent binding to structural proteins of the extracellular matrix [16] and reaches the blood stream indirectly through the lymphatic system. Later, exposure to specific allergen leads to cross-linking of the IgE on sensitized cells, resulting in degranulation and secretion of pharmacologically active mediators that act primarily at the area of the allergen exposition (Fig. 3).

Although IgE was detected in preparations destined for IVIg injections [9, 17, 18], not the same scrutiny was applied to testing ScIg preparations for the IgE content. Here we report such testing for the first time. Literature reports deal with Endobulin^®^ and Sandoglobulin^®^ [9] or Bioglobulin^®^ [18] where IgE was found in all series of IVIg commercial preparations, in the amounts from 34.5 to 105 IU/ml [17] or class 1 [9]. A higher level between 138 and 232 IU/ml or class 2 was found in Subcuvia^®^ (Tables 2, 3, 4), but number of IgE units determined per gram of protein were comparable (690–2100 IU/g in 5% IVIg solution and 862.5–1450 IU/g in our 16% ScIg solution).

The transfusion of IgE with ScIg was an excellent opportunity to observe IgE absorption and distribution (Fig. 2). This finding is compatible with the short serum half-life of IgE and much longer in tissue compartment [19–21] (Fig. 2). Unlike cases described in the publications dealing with passive transfer of IgE with blood products [19, 20], in our report patients were immunodeficient (showed low expression of Fc R—see below), did not receive single transfusion from one donor, but subcutaneous replacement therapy (in contrast to intravenous frozen plasma or platelet infusions) with various batches of Subcuvia (Table 2; Fig. 2). Therefore, their cumulative exposure to IgE was higher and longer (Fig. 2).

Specific immunoglobulins may coat Fc-receptors of various immune cells. Such phenomenon is well documented for IgE, primarily described as passive cutaneous anaphylaxis or the Prausnitz–Küstner (PK) reaction. The PK is a local hypersensitivity reaction induced by intracutaneous injection of serum from a hypersensitive individual into a healthy person. When after 24 h since the commencement of the exchange the antigen to which the donor is allergic, is injected for the second time, the wheal-and-flare response occurs. It is characteristic that “immediate” type allergic reaction is observed at the time when the specific antigen is injected (or inhaled or ingested as in the case of our patient) (Fig. 3). The classic model was for the first time observed in our study after injection of ScIg containing IgE (Fig. 1). After ScIg local hypersensitivity reaction is observed (as PK reaction), unlike a systemic reaction observed after transfusion of IVIg, frozen plasma or platelet (passive anaphylaxis) [9, 18–20]. So far, positive wheal/flare reactions to various IVIg and ScIg preparations have been interpreted as active IgE-mediated sensitivity to immunoglobulins [3]. Our observation with early and late phase reaction during ScIg infusion (Fig. 1) together with IgE content in Subcuvia^®^ give the alternative explanation. Patients consumed normal food products (without milk, egg, pork elimination), sera of one patient were positive for Candida antigen before CVID diagnosis (i.e. −22 days, data not shown) [22]. Candida-specific IgE infusion i.e. passive immunization at 0.45 kUA/l (Table 4) may be one cause of PK reaction, presented here (Fig. 1).

It is described that serum IgE levels regulate FcεRI expression on mast cells and basophils: half-life of FcεRI on cell surface is stabilized by binding IgE [23]. The regulation is independent of the disease [24] so IgE-deficiency in CVID coexists with low Fc RI expression. Such low expression may be corrected by injecting exogenous IgE (IgE at 5 μg/ml resulted in 32-fold elevation (at day 4) in FcεRI expression) [25]. After temporary increase of IgE level in +2 days after ScIg transfusion significant decrease and negative result of serum IgE were observed, analogously at day 4, i.e. before the next transfusion (Fig. 2). Furthermore, in response to IgG immune complexes, basophils release the platelet-activating factor, a major biochemical mediator of the systemic anaphylaxis (Fig. 3) [26]. An additional hypersensitivity mechanism is probable when rapid antigen binding by IgG after infusion of immunoglobulin containing rheumatoid factor, which caused an Arthus-type reaction (type III, vasculitis), complement activation, anaphylatoxin release followed by basophil or mast cells degranulation and subsequently intensification of hypersensitivity (Fig. 3). On the other hand, basophils express inhibitory Fc RII (CD32) that are the source of FcεRI + FcγRII co-stimulation and inhibition of Syk-dependent histamine release [26, 27]. Finally, these mechanisms may be the source of the observed in our study serum IgE fluctuation in spite of the continuous IgE injection with subsequent doses of ScIg (timeline presented in Fig. 2a).

## Conclusions

Our observation of passive transfer of hypersensitivity indicates that rigorous blood donor screening for allergies should be considered and the blind-spot testing for IgE as part of ScIg quality control should be performed. Furthermore, until now late manifestation of immediate type hypersensitivity after ScIg (positive ICT remains detectable for weeks to months (Fig. 2) has not been described in medical literature nor as product characteristics. Therefore, there are no strict safety regulations for home-based self-administration of immunoglobulins. The ScIg are non-bioequivalent products (Table 2; Fig. 2) and are misnamed as a medication. IgE has a short serum half-life (i.e. days, Fig. 2a), but after binding to Fc it resides in tissues for months (Fig. 2). Due to prolonged risk of anaphylaxis after IgE passive transfer, switching from in-hospital administration to home self-administration should be done with an extreme care, and screening for allergy by SPT/ICT is recommended. In pharmaceutical process testing of immunoglobulin products for IgE content is not part of routine practice but could be considered. Testing for newly-acquired IgE sensitivities should be considered in patients receiving IgE-containing immunoglobulin products, particularly in those patients who develop new allergic sensitivities, and would be an interesting area to study prospectively (Table 5).

**Table 5:** Difference between immunoglobulins (ScIg, IVIg) and typical drugs

Feature	Drug/medicine	Immunoglobulin
Source	Chemical (for example catalytic, enzymatic) reaction “IN VITRO”	Plasma obtained from blood donors^a^ “IN VIVO”
Chemical composition, dose	Known (standardization following pharmacopeia) Dose [g] describe active compound content	Not precisely defined Protein (in reality proteins complex) Dose [g] describe total protein content
Clinical pharmacology		
Mechanism of action	Defined (by strict receptors)	Multifactorial (depends on antigenic specificity)
Elimination and half-life (T½)	Renal intestinal etc. after easy diffusion from tissue compartment Strictly defined T½	Not known Serum T½ does not reflect immunoglobulin elimination, but FcR opsonization^b^
Unpredictable adrs		
Immunogenicity	Usually low (in general haptenic formula)	Pathological anti—IgA^c^
Immunoreactivity (immune reaction to pharmaceutical product)	Rare, but ever probable (e.g. benzylpenicillin allergy)	Physiological tolerance or antiidiotypic immune response^d^

Immunoglobulins are a pharmaceutical product with unique technology, contrary to bio-synthetic drugs

^a^Immunoglobulin preparation is plasma fractionation: plasma is obtained in accordance with WHO guidelines from at least 1000 donors

^b^After immunoglobulin distribution to tissue compartment FcR opsonization occurs (see Figs. 2a, 3). It blocks inverse diffusion to serum

^c^In clinical practice immunoglobulins are used as replacement therapy in patients with primary and secondary immunodeficiencies. Lack of active immune response after antigenic stimulation (e.g. vaccination) in such patients is one crucial mechanism of low immunogenicity. Contrary to CVID, patients with selective IgA deficiency may produce IgG that reacts with IgA in immunoglobulins (product characteristic of many immunoglobulins as well as Subcuvia^®^ does not contain essential contraindication—selective IgA deficiency)

^d^When immunoglobulin is used in immunomodulatory therapy (autoimmune disease) the immune response to immunoglobulins and hyperreactivity may be observed. For example Rheumatoid factor from patients with autoimmune disease and IgG from pharmaceutical product

## Abbreviations

PAD: primary antibody deficiencies
CVID: common variable immunodeficiency
ScIg: subcutaneous immunoglobulin G
IVIg: intravenous immunoglobulin G
ADRs: adverse drug reactions
T½: half-life
SPT: skin prick test
ICT: intracutaneous test
LPR: long phase reaction
EPR: early phase reaction
RF: rheumatoid factor
HSCT: hematopoietic stem cell transplantation
U/l: units per litre
kU/l: kilo unit per litre (1 kU/l or IU/ml is equal to 2.4 ng/ml)
AT: Arthus-type reaction
CT: coombs tests
PK: Prausnitz–Küstner
