# Correction to: Passive blood anaphylaxis: subcutaneous immunoglobulins are a cause of ongoing passive anaphylactic reaction

**DOI:** 10.1186/s13223-018-0302-5

**Published:** 2018-10-05

**Authors:** Przemyslaw Zdziarski, Andrzej Gamian, Jacek Majda, Agnieszka Korzeniowska‑Kowal

**Affiliations:** 1 Department of Clinical Immunology, Lower Silesian Center for Cellular Transplantation, POBox 1818, 50-385 Wrocław-46, Poland; 2 Department of Immunology of Infectious Diseases, Hirszfeld Institute of Immunology and Experimental Therapy, Polish Academy of Sciences, POBox 1818, 50-385 Wrocław-46, Poland; 3 Department Laboratory, 4th Military Teaching Hospital, Wrocław, Poland

## Correction to: Allergy Asthma Clin Immunol (2017) 13:41 10.1186/s13223-017-0213-x

Upon publication of the original article [1], the authors reported the following funding information was omitted: Publication supported by Wroclaw Centre of Biotechnology, programme The Leading National Research Centre (KNOW) for years 2014–2018.
